# Museums of London

**Published:** 1853-03

**Authors:** 


*Museums of London.*—The following account of the Museums of London, is interesting in its reference to the lives and labors of the distinguished men originating them. The account is written by a correspondent of the *Dublin Medical Press*, author of a series of letters in that able print, entitled *Medical Life in London*—letters, as severe as racy, from which we have heretofore copied some extracts.—Editor Buffalo Med. Journal.

"Our greatest museum in London was founded by an Irishman, Sir Hans Sloane; the great College of Surgeons' collection by a Scotchman, John Hunter; while at the College of Physicians, poor Harvey was nine years illustrating his doctrine of the circulation, with preparations, all in vain. 'He fell mightily in practice,' we are told; and in London it was believed

'that he was crack-brained, and all the physicians were against him.' His 'therapeutic way' was not admired, and he was left on the high road to starvation but for King Charles. These facts, at least, should make us a little modest. It is good for us occasionally to think over the lives of such men. Hunter dying in debt, and his magnificent collection going begging, refused by this College of Physicians, and grudgingly received into its present situation. Sir Hans Sloane giving away thirty years' income as charity, but he himself now half forgotten.

"Sir Hans Sloane was born, according to some documents in the Library of his splendid collection at the British Museum, in the County Down, Ireland, at a place called Killeagh, April 16, 1660. He stated in his will that the collection he was bequeathing the nation was richly worth £80,000; it contained 200 volumes of dried plants in the form of a *hortus siccus*, 30,000 minerals and other specimens of great interest in natural history, with a library of fifty thousand volumes, and 3566 very rare manuscripts. There are two pictures of this great and talented Irishman in the museum. One would like to see them more generally known. It appears from the little written of Sir Hans Sloane, that it was in Ulster, in Ireland, he first imbibed that love of scientific pursuits that have since rendered his name, and we fear only his name, illustrious; in France and Ireland, in fact, was laid the foundation of his great museum. Like Hunter, Mozart, Goldsmith, Hadyn, Johnson, and a legion of other great men, we find Sloane, in early years, struggling a good deal with adversity. Before he was of age he had several severe attacks of hæmoptysis, which threatened him with an early grave, like Laennec, Bichât, and others; death, however, spared him till he had done his work. Stahl, Ray, and another great countryman, Robert Boyle, were then in the ascendant, all of whom were known to Sloane, and helped to form his mind. And yet who thinks, amid the winged bulls from Nineveh, and the magnificent collection crowded at present in the huge building of the British Museum, of Sir Hans Sloane or Boyle. We think of Watt whenever we see a steam engine, because, with one happy thought, he has made it the last wonder of the world in a utilitarian point of view: the great workers in the mine of abstract science and philosophy, we disregard. In 1683, young Sloane set off for France, and there seems to have been delighted with the botanical collections and lectures of Tournefort, and spent a year collecting plants for the museum. We find him next going out to the West Indies, a young man, under 30, physician to the Duke of Albemarle, and in spite of many crosses and disappointments, still further adding to his specimens 800 species of rare and valuable tropical plants. These two collections form the first nucleus of the British Museum. It seems all this time he never forgot his native country, Ireland, and we should in all probability have those specimens now in Trinity College, Dublin, and the British Museum (which would be a great blessing) quite a different institution, but that Sloane got married to a very rich wife, and settled permanently in London. In 1693, he became secretary of the Royal Society, and in 1727 was appointed physician to the king, and succeeded the great Newton as president of the Royal Society. George I. made him a baronet, and he died, at the age of 93, in 1753.

"Of the life of the great founder of the Hunterian Museum we need say nothing; the facts of the eventful biography of Hunter are among the household words of the profession. He, too, was looked upon as an innovator, like Harvey, and fought his way to his position among the greatest men that ever lived. Hunter or Harvey have no monuments in brass or marble in London; but they want none: their memories and great acts are enshrined in every good man's soul, without the empty nonsense of colleges. Our American and other friends walking through the galleries of the Museum, should recollect that it was Sir J. Banks and Lord Auckland rescued it from destruction; that poor Hunter's worldly chattels were all sold for debt—for debts incurred for putting the museum together; that the College of Physicians refused the museum as a gift, and that under certain favor, it was offered a domicile by the College of Surgeons, whose income, from getting the hard-earned work of Hunter's life, was doubled after a little, so that in 1833, it was said to be over £10,000 a year, and £66,000 in hands; the government gave £30,000 to build the museum, and £15,000 for the preparations. With all this money, or a tithe of it, in Dublin, with another Sir Hans Sloane, Macartney, or Carmichael, what miracles might not Ireland perform. Poor Hunter's life seems to have been one battle. He set out early, under the guidance of his brother, with whom he soon fell out; in 1753 he goes to Oxford, laughs at Latin and Greek, and a little after we find him battling with the Monros and his brother. His next encounter was with Pott—a sort of Syme of those days, that every one thought it correct to have a tilt with. Our intention, however, at present, is to say something of the museum, and wish peace to the troubled shade of its great founder.

